# Supplementary material for: Friend, Not Foe: Lowered Tissue Reactivity to Long‐Term Polyimide Implants
Source: Adv Sci (Weinh). 2026 Apr 15:e00028. Online ahead of print. doi: 10.1002/advs.202600028 (PMC13334871; doi:10.1002/advs.202600028)
Supplement: Supplementary file 1 — Supporting File: advs75276‐sup‐0001‐SuppMat.docx. [file ADVS-9999-e00028-s001.docx]

**Supplementary Figures**

**
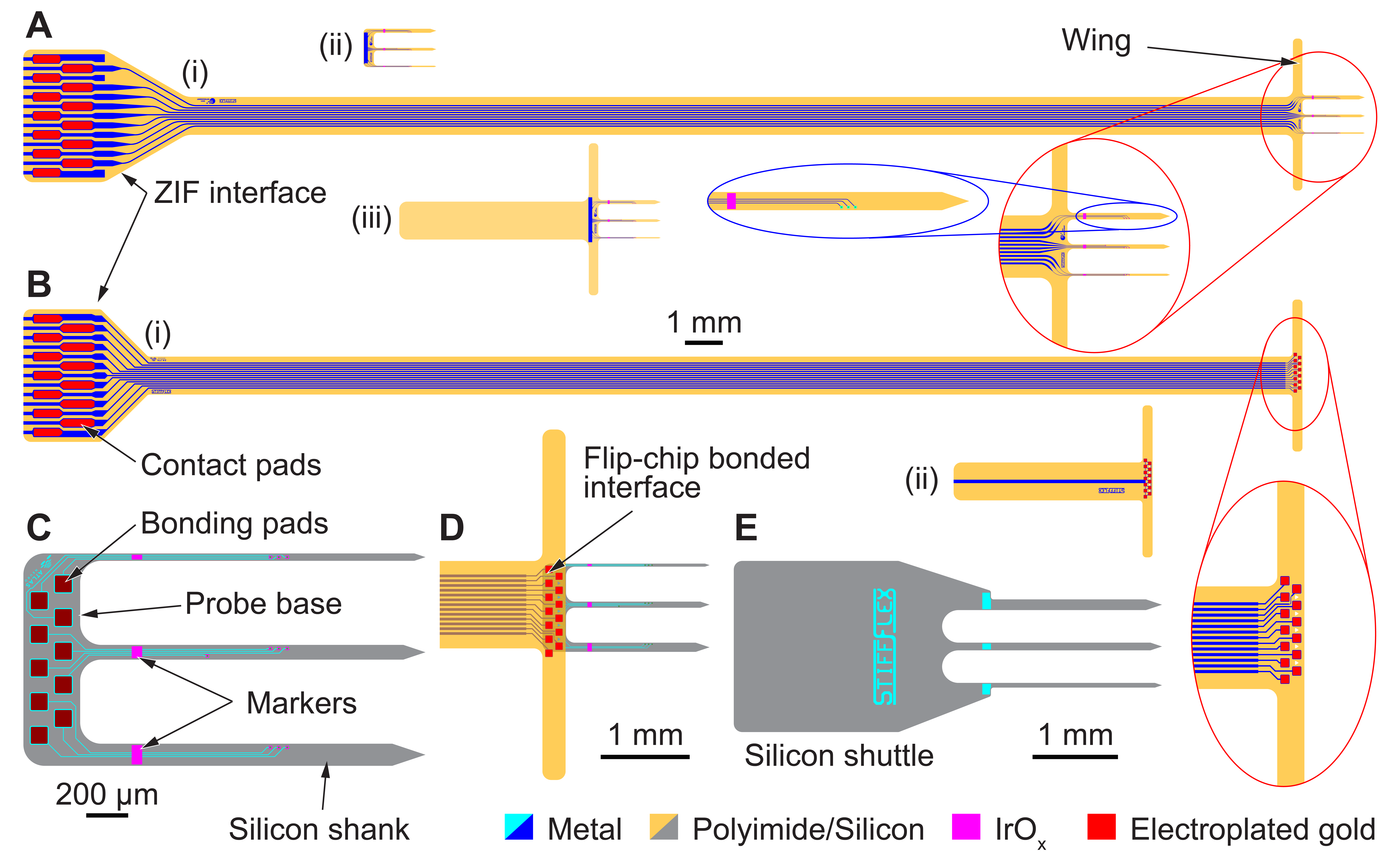
**

**SUPPL. FIGURE 1**

Neural probe designs. (**A)** Polyimide probes and test structures with three implantable shanks: (i) Functional probe with a 28-mm-long cable connected to a ZIF interface. The wing structure is used to fix the probe after insertion to the skull with dental cement; (ii) floating implant with a slim base interconnecting the probe shanks, and (iii) tethered implant with a short PI strip mimicking the cable in (i). **(B)** Interconnecting cables for (i) the Si-based probe used for neural recording and (ii) dummy cable with a short PI strip mimicking the cable in (i). **(C)** Silicon probe with 10 bonding pads to which the cable from (B-i) or cable dummy from (B-ii) are flip-chip bonded. **(D)**Flip-chip bonded interface between the Si-based neural probe and the PI-based cable. **(E)** Silicon-based insertion shuttle with a larger base to facilitate the handling of the probe.


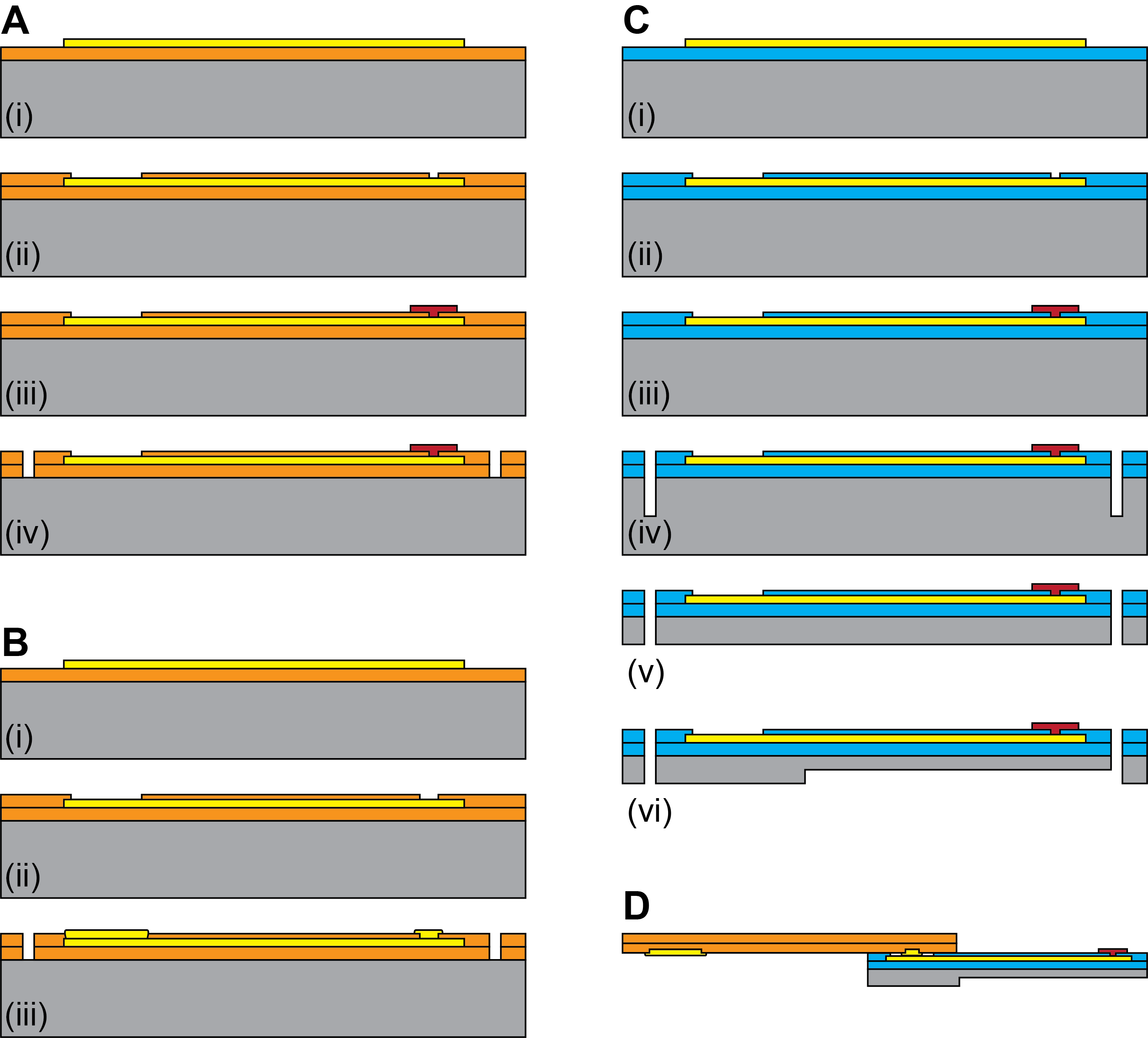


**SUPPL. FIGURE 2**

Fabrication process. (**A)** Fabrication of PI-based neural probe: (i) Spin-coating of the first polyimide layer, and deposition and patterning of metallization (100 nm Pt) using sputtering and a lift-off process, (ii) spin-coating of the second polyimide layer and RIE to open recording sites (electrodes) and contact pads, (iii) reactive sputter deposition and lift-off patterning of IrO_x_ electrode coating on top of the polyimide layer, and (iv) patterning of neural probe contour using RIE. **(B)** Fabrication of polyimide interconnecting cable: (i) Spin-coating of first polyimide layer, and deposition and patterning of metallization (100nm Pt) using sputtering and a lift-off process, (ii) spin-coating second polyimide layer and RIE to open contact and bonding pads and definition of the cable contour, and (iii) electroplating of contact and bonding pads with gold. **(C)** Fabrication of silicon probes: (i) Deposition of the first passivation layer containing a layered stack of SiO_x_ and Si_x_N_y_ using PECVD, and deposition and patterning of metallization (100 nm Pt) using sputtering and a lift-off process, (ii) deposition of second passivation layer stack of SiO_x_ and Si_x_N_y_ using PECVD and RIE patterning of recording sites and contact pads, (iii) reactive sputter deposition and lift-patterning of IrO_x_ electrode coating on top of the SiO_x_/Si_x_N_y_ passivation layer, (iv) patterning of probe contour by RIE and bulk micromachining of silicon using DRIE, (v) wafer grinding to thin down the silicon substrate, and (vi) selective DRIE to thin down the probe shanks using the EBAG process. **(D)** Silicon probe assembly: Flip-chip bonding of the silicon probe to the polyimide interconnecting cable.

**
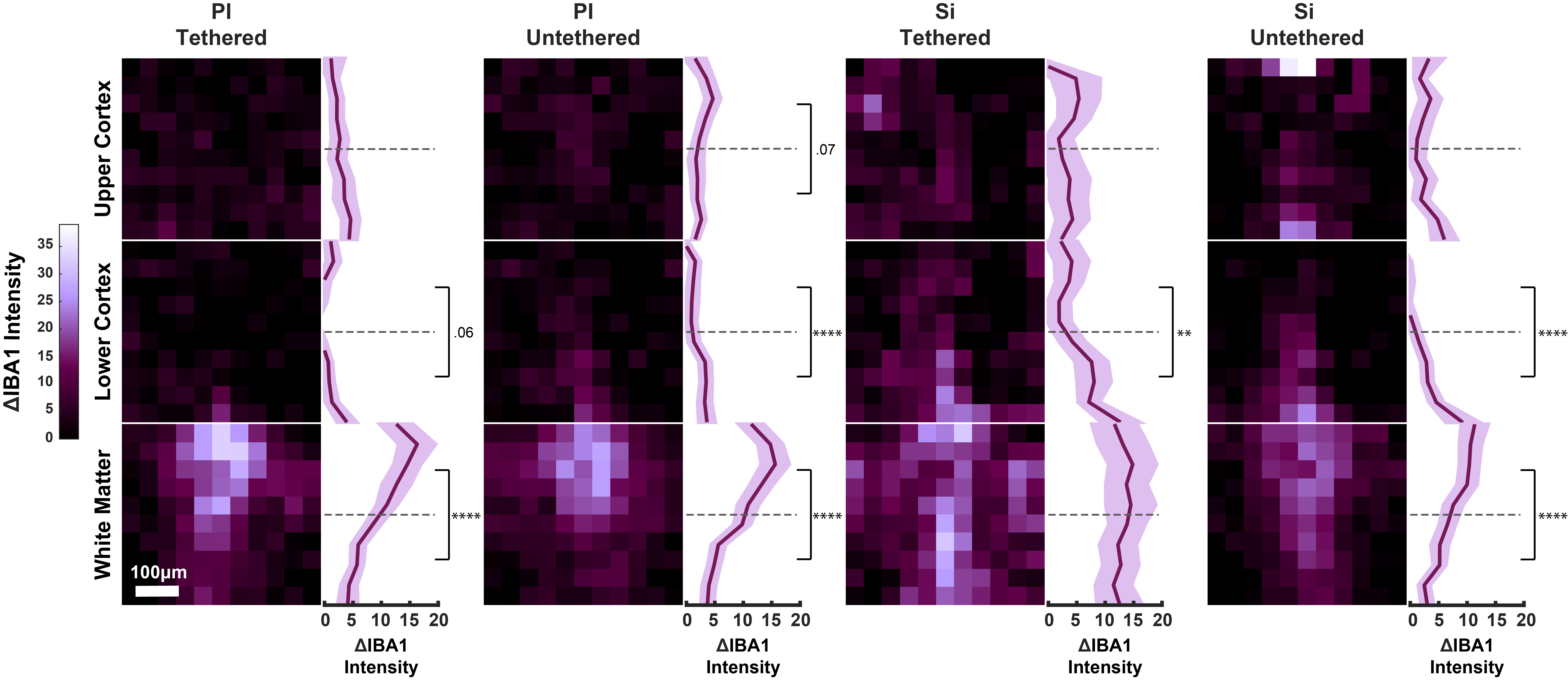
**

**SUPPL. FIGURE 3**

Microglial depth profile for the tethered and untethered silicon and polyimide probes. Scale bar = 100 μm. PI = Polyimide; Si = Silicon. Stacked heatmaps correspond to upper cortex (top), lower cortex (middle) and white matter (bottom). Line graphs display the average ΔIBA1 value across cortical depth. Shaded areas represent SEM. Significant differences of signal intensities in upper and lower areas within each ROI are marked. All conditions display an increased ΔIBA1 intensity in the white matter. * p < .05, ** p < .01, *** p < .001, **** p < .0001. Created with BioRender.com

**
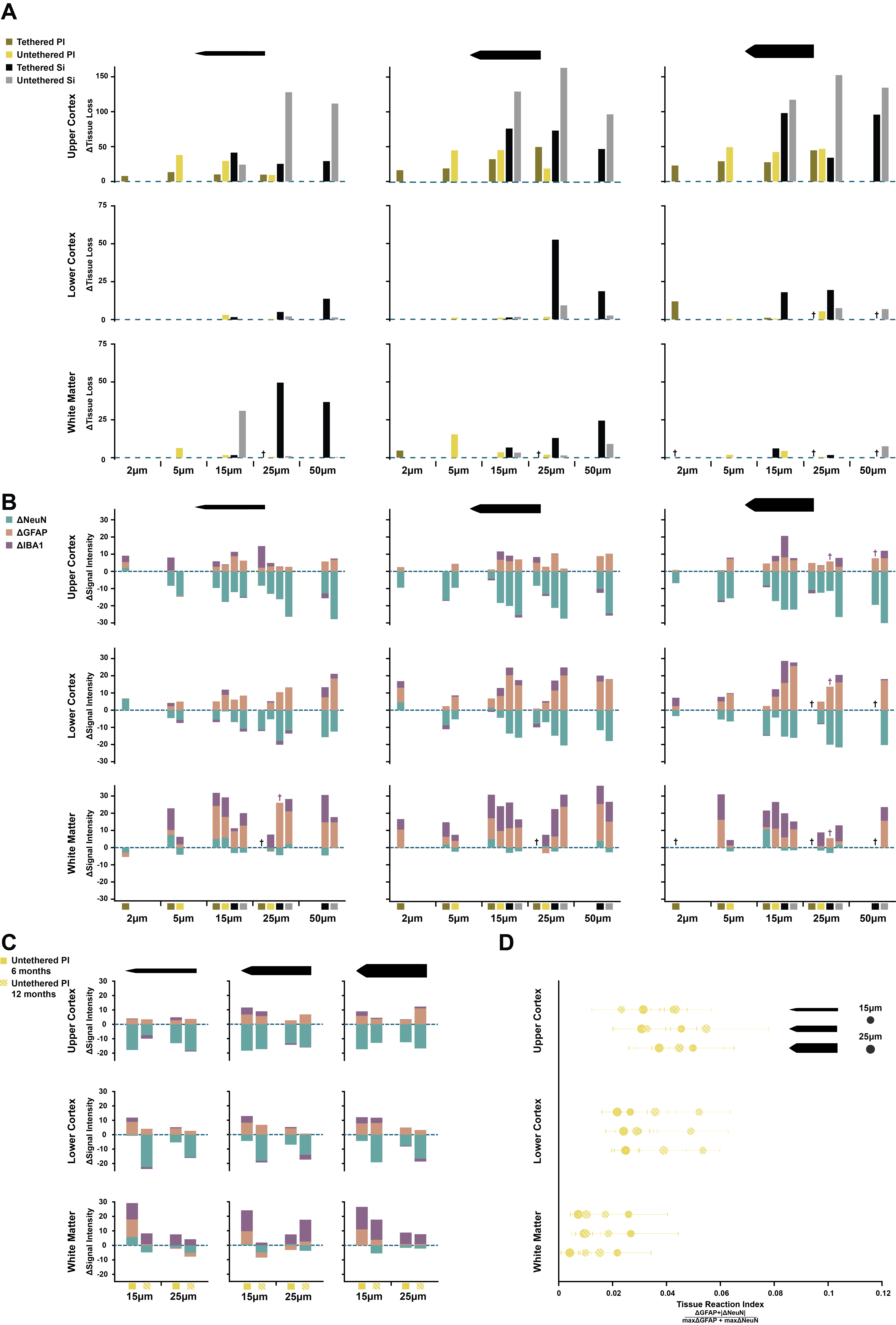
**

**SUPPL. FIGURE 4**

Tissue responses across all experimental conditions. **(A)** ΔTissueLoss caused by the various probes with a shank of 35 (left), 70 (middle column), 105 µm width (right) in upper cortex (top), lower cortex (middle row) and white matter (bottom). **(B)** ΔNeuN (neuronal loss), ΔGFAP (astrocytic response) and ΔIBA1 (microglial response), in the same format as panel A. The colors below the x-axis represent the various material and tethering combinations. Black plusses mark missing datapoints for conditions with one sample or less. Colored plusses mark missing datapoints in a specific staining. **(C)** Untethered polyimide probes implanted for 6 or 12 months of shanks with a width of 35 (left), 70 (middle column) and 105 µm shank width (right column). **(D)** Idx_TissueReaction_ for untethered polyimide probes implanted for 6 and 12 months.

**
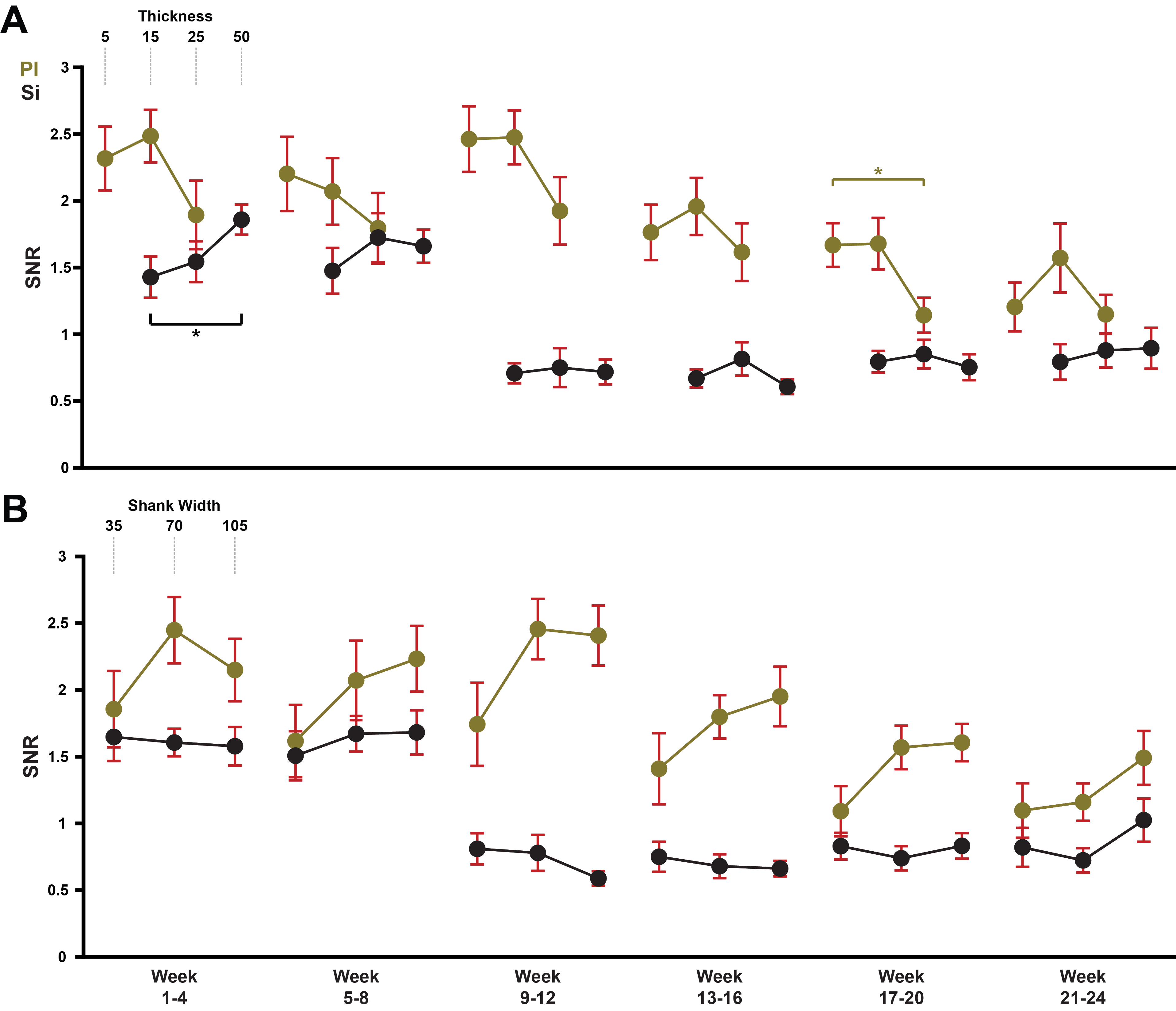
**

**SUPPL. FIGURE 5**

Average signal-to-noise (SNR) ratio for electrodes on shanks with different thicknesses and widths in 4 week time-bins. PI = Polyimide; Si = Silicon. Error bars, SEM. **(A)** Influence of probe thickness on SNR. Polyimide probes had a thickness of 5, 15, or 25 µm and silicon probes were 15, 25 or 50 µm thick. **(B)** SNR of electrodes from shanks with a width of 35, 70 or 105 µm. * p < .05.

**
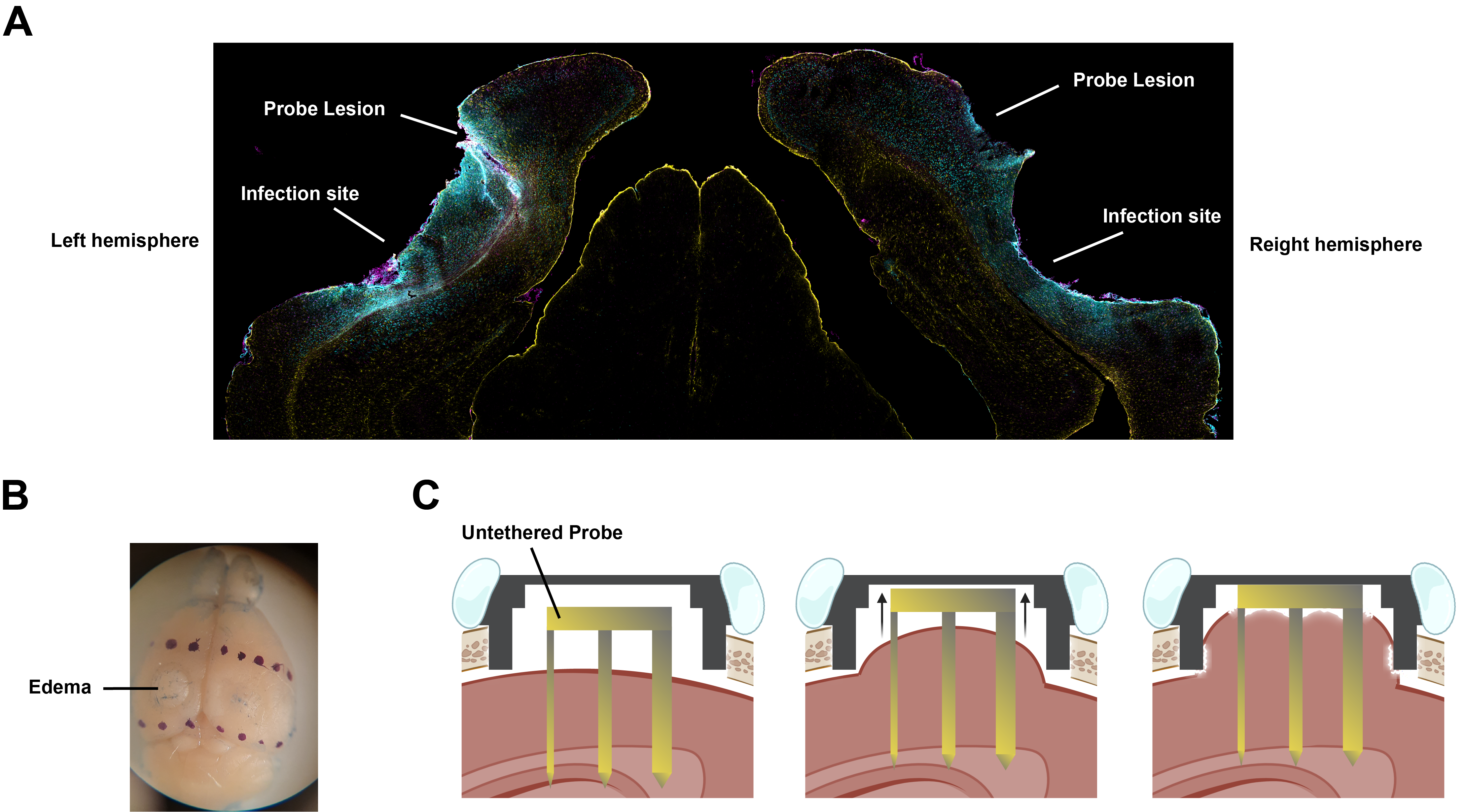
**

**SUPPL. FIGURE 6**

Observed adverse effects on the brain tissue. **(A)** Excerpt of full brain image of one of the 2 animals implanted with functional polyimide probes. There was an infection close to the surgical site in both hemispheres, causing regional gliosis. **(B,C)** Effect of the surgical method used to implant untethered probes. We made a 3 mm circular craniotomy and closed the skull with a stainless-steel cap. **(B)** Example brain with edema at the site of the implantation in the left hemisphere. **(C)** Illustration of the possible negative impact of the cavity above the cortex. In some cases, the cortex and the probe were displaced into the cavity in the direction of the inner roof of the cap. Created with BioRender.com
